# Tumor or Inflammation? How Multi-Modality Image Fusion and Shear Wave Elastography Can Help Solve the Diagnostic Dilemma

**DOI:** 10.3390/diagnostics16152300

**Published:** 2026-07-23

**Authors:** Efstathios T. Detorakis, George Bontzos, Eleni E. Drakonaki

**Affiliations:** 1 Department of Ophthalmology, University Hospital of Heraklion, 715 00 Crete, Greece; gbontzos@hotmail.gr; 2 Department of Anatomy, University Hospital of Heraklion, 715 00 Crete, Greece

**Keywords:** differential diagnosis, masquerade syndromes, intraocular lesions

## Abstract

Differentiating intraocular tumors from inflammatory conditions remains a major diagnostic challenge in ophthalmology, particularly in masquerade syndromes where clinical and imaging features overlap. Despite advances in ocular imaging, no single modality provides sufficient diagnostic specificity. This narrative review synthesizes current evidence on optical imaging modalities, ultrasonography, and elastography, focusing on their complementary roles in intraocular disease characterization, with emphasis on shear wave elastography (SWE) within a multimodal framework. Conventional imaging provides partial insights: OCT and ultrasonography define structural features, while angiographic techniques assess vascular behavior. Elastography adds a biomechanical dimension by enabling in vivo assessment of tissue stiffness. Malignant lesions typically exhibit increased stiffness and heterogeneity, whereas inflammatory processes show more variable profiles, yet overlap exists. Integrating structural, vascular, and biomechanical data improves pattern recognition and diagnostic confidence. A tri-axis fusion model and a practical diagnostic workflow are proposed to support clinical decision-making. Multi-modality image fusion represents a shift toward hybrid multi-dimensional tissue characterization in ophthalmology. The addition of elastography may assist in the differential diagnosis process between intraocular tumors and inflammatory conditions. Further standardization, validation, and integration with artificial intelligence are required for routine clinical implementation.

## 1. Introduction

Tumors and inflammatory conditions represent classic “masquerade” entities in ophthalmic diagnostics. The traditional clinical signs of inflammation—*tumor*, *dolor*, *calor*, *rubor* (swelling, pain, increased tissue temperature, and redness)—first described by Celsus in antiquity and later complemented by Virchow in the 19th century with the addition of *functio laesa* (impaired function), are frequently encountered in ocular inflammatory diseases. However, many of these predominantly inflammatory features may also be produced by intraocular neoplastic conditions, which can clinically mimic inflammation and give rise to so-called “masquerade syndromes” [1,2].

The intraocular environment is an anatomically confined space containing highly specialized and delicate structures. As a result, obtaining tissue for histopathological confirmation—either through vitreoretinal surgery or with less invasive techniques such as fine needle aspiration (FNA)—is often diagnostically uncertain and associated with significant risks. These include iatrogenic damage to critical structures such as the retina, optic nerve head, or crystalline lens, as well as the potential for tumor cell dissemination. In certain malignancies, such as retinoblastoma, extraocular tumor spread through ocular wall entry sites may alter tumor staging and carry potentially life-threatening consequences [3]. Liquid biopsy techniques, using either systemic fluids (e.g., peripheral blood) or ocular samples (aqueous or vitreous humor), have emerged as promising diagnostic tools. Nevertheless, their current diagnostic yield for intraocular tumors remains limited, particularly when based on peripheral blood, while ocular fluid sampling carries an inherent risk of tumor dissemination due to the need for globe penetration [4].

In recent years, advances in ophthalmic imaging—including optical coherence tomography (OCT), OCT angiography, laser scanning imaging, and ultrasonography—have offered new opportunities for the noninvasive assessment of intraocular pathology. Ultrasonographic techniques may be performed using dedicated ophthalmic systems (A-scan and B-scan for the posterior segment, Ultrasound Biomicroscopy-UBM for the anterior segment) or radiology-grade platforms, which enable additional modalities such as color Doppler ultrasonography and ultrasound elastography. The latter, either in the form of strain elastography (SE) for more superficial tissues or in the form of shear wave elastography (SWE) for deeper tissues can be used to obtain qualitative (SE) or quantitative (SWE) information on tissue rigidity and biomechanical behavior. Together, these techniques provide complementary structural, vascular, and biomechanical insights. Despite these developments, a significant diagnostic gap persists in cases where clinical and imaging findings lie at the intersection of inflammation and neoplasia. Importantly, the creation of hybrid images by fusion between imaging scans obtained by different modalities can enhance the diagnostic potential through the incorporation of different properties from each modality in the hybrid image. As each modality offers information from a specific standpoint—morphological, vascular, or biomechanical—there is a growing need to integrate data from multiple sources in order to more comprehensively characterize suspicious intraocular lesions [5].

This narrative review examines the role of multi-modality imaging fusion, with particular emphasis on shear wave elastography, as a complementary diagnostic approach for differentiating intraocular tumors from inflammatory conditions. Illustrative clinical cases are presented to highlight practical applications, interpretative strategies, and potential diagnostic patterns relevant to uveitis and vitreoretinal specialists.

## 2. Current Light-Dependent Imaging Modalities in Intraocular Disease

### 2.1. Color Fundus Photography (CFP)

Color fundus photography (CFP) remains the cornerstone for documentation and longitudinal assessment of retinal and optic nerve pathology owing to its accessibility, reproducibility, and faithful color representation of the ocular fundus [5]. In inflammatory diseases, CFP demonstrates retinal whitening, vasculitis, and chorioretinal scars, whereas in ocular tumors it documents lesion size, pigmentation, margins, and associated retinal alterations, facilitating follow-up [6]. However, its two-dimensional nature, lack of depth resolution, and inability to assess vascular or biomechanical characteristics limit its value in differentiating inflammatory from neoplastic lesions. Therefore, CFP should be regarded primarily as a complementary structural imaging modality within a multimodal diagnostic approach [6].

### 2.2. Fundus Autofluorescence (FAF)

Fundus autofluorescence (FAF) provides functional information on retinal pigment epithelium (RPE) integrity by detecting lipofuscin-related autofluorescence [7]. In inflammatory disorders, FAF identifies areas of RPE stress, active inflammation, or atrophy, whereas in ocular tumors it highlights secondary RPE alterations and tumor-associated lipofuscin. Although useful for detecting metabolic disturbances, similar autofluorescence patterns may occur in both inflammatory and neoplastic conditions, limiting diagnostic specificity. Consequently, FAF is most informative when interpreted together with structural and vascular imaging modalities [7].

### 2.3. Fluorescein Angiography (FFA)

Fluorescein angiography (FFA) remains an important modality for assessing retinal vascular integrity, leakage, and blood-retinal barrier disruption [8]. It is particularly useful for evaluating inflammatory activity through leakage, vasculitis, and capillary non-perfusion, while intraocular tumors may demonstrate intrinsic vascularity and characteristic leakage patterns. However, considerable overlap exists between inflammatory and neoplastic angiographic findings, and FFA provides limited information on the choroidal circulation or tissue composition. Consequently, its principal contribution lies in complementing structural imaging within a multimodal diagnostic strategy [8].

### 2.4. Indocyanine Green Angiography (ICGA)

Indocyanine green angiography (ICGA) enables superior visualization of the choroidal circulation and is particularly valuable for diseases involving deeper ocular structures [8]. It provides complementary information on choroidal vascular abnormalities, inflammatory involvement, and tumor vascularization. Nevertheless, ICGA does not directly characterize tissue composition or biomechanics and therefore cannot reliably distinguish inflammatory from neoplastic lesions when used in isolation. Its greatest value lies in combination with structural and biomechanical imaging modalities [8].

### 2.5. Laser Scanning Ultra-Widefield Imaging

Laser scanning ultra-widefield (UWF) imaging expands retinal visualization to approximately 200°, allowing detection of peripheral pathology that may be missed by conventional fundus photography [9,10]. This is particularly useful in both inflammatory disorders and intraocular tumors, where peripheral vasculitis, satellite lesions, or multifocal involvement may influence the differential diagnosis. Despite its wide field of view, UWF imaging remains primarily a structural modality without direct biomechanical information and should therefore be interpreted alongside OCT, angiography, and ultrasonography [10].

#### 2.5.1. Optical Coherence Tomography (OCT)

Optical coherence tomography (OCT) provides high-resolution cross-sectional visualization of retinal and choroidal microstructure and has become indispensable for evaluating intraocular disease [11]. It identifies lesion morphology, retinal edema, subretinal fluid, and disruption of retinal architecture, thereby contributing significantly to the distinction between inflammatory and neoplastic processes. However, OCT remains primarily a structural imaging technique and provides little direct information regarding vascular function or tissue biomechanics. Accordingly, its diagnostic value is maximized when integrated with complementary vascular and ultrasonographic modalities [11].

#### 2.5.2. Optical Coherence Tomography Angiography (OCT-A)

Optical coherence tomography angiography (OCT-A) non-invasively visualizes retinal and choroidal microvasculature by detecting motion contrast from circulating erythrocytes [12,13]. It complements conventional angiography by providing depth-resolved vascular imaging without dye administration and is useful for assessing microvascular alterations associated with both inflammatory and neoplastic diseases [14]. Nevertheless, motion artifacts, segmentation errors, and reduced sensitivity to slow blood flow may limit interpretation, while vascular findings alone are often insufficient to distinguish tumors from inflammation because considerable overlap exists between the two entities [15].

#### 2.5.3. OCT Elastography

OCT elastography extends conventional OCT by providing information on tissue biomechanics through assessment of local tissue deformation [16]. Its exceptionally high spatial resolution offers the potential to characterize biomechanical changes within retinal and choroidal tissues and may eventually complement ultrasound elastography in differentiating inflammatory from neoplastic lesions. However, because of its limited penetration depth and its largely experimental status, OCT elastography currently remains a promising research technique rather than a clinically established diagnostic modality [16].

## 3. Ultrasonography in Intraocular Diagnostics

### 3.1. A-Scan and B-Scan Ultrasonography

A-scan and B-scan ultrasonography are best considered complementary techniques, typically employing frequencies of 8–12 MHz. While A-scan provides quantitative information on tissue reflectivity, B-scan offers spatial and morphological context. Together, they enable a more comprehensive assessment of intraocular pathology than either modality alone. However, both techniques have limitations [17]. Ultrasound resolution is lower than that of OCT, limiting the ability to visualize fine retinal microstructure. Interpretation can be operator-dependent, and image quality may be affected by probe positioning and patient cooperation. Additionally, while ultrasonography provides indirect information about tissue composition through reflectivity patterns, it does not offer direct insight into vascular function or biomechanical properties. In modern ophthalmic practice, A- and B-scan ultrasonography remain essential, particularly in cases where optical imaging is limited or inconclusive [18]. They are often used in conjunction with other modalities, including OCT, angiography, and, increasingly, Doppler-based techniques, to provide a more complete understanding of intraocular disease [17].

### 3.2. Ultrasound BioMicroscopy (UBM)

Ultrasound biomicroscopy (UBM) is a high-frequency ultrasound technique (typically 35–50 MHz) designed for detailed imaging of the anterior segment. By utilizing higher frequencies than conventional B-scan ultrasonography, UBM provides superior spatial resolution, allowing precise visualization of structures such as the cornea, anterior chamber, iris, ciliary body, and anterior sclera. This makes it particularly valuable in cases where optical imaging is limited or obscured, including eyes with corneal opacity, hyphema, or dense cataract. In the context of intraocular tumors and inflammatory conditions, UBM plays a key role in the evaluation of anterior segment lesions, including iris and ciliary body masses. It enables accurate assessment of lesion size, extent, internal reflectivity, and involvement of adjacent structures, which may not be adequately visualized with slit-lamp biomicroscopy or anterior segment OCT. Inflammatory processes, such as anterior uveitis or ciliary body edema, may present as diffuse thickening or structural distortion, whereas neoplastic lesions are more likely to appear as localized masses with defined borders. However, as with other structural imaging modalities, overlap in imaging features may limit specificity [19].

Despite its high resolution, UBM has several limitations. Tissue penetration is relatively limited due to the high-frequency ultrasound used, restricting visualization to the anterior segment. The technique is contact-based and requires immersion or a coupling medium, which may reduce patient comfort and limit routine use. Additionally, UBM provides primarily structural information and does not directly assess vascular or biomechanical properties. Within a multimodal imaging framework, UBM serves as a complementary tool for high-resolution anterior segment assessment. When integrated with posterior segment imaging, Doppler techniques, and elastography, it contributes to a more comprehensive characterization of intraocular pathology, particularly in cases involving the ciliary body or anterior uveal tract [19].

### 3.3. Triplex Ultrasonography (B-Scan, Color Doppler, Spectral Doppler)

Triplex ultrasonography combines conventional B-scan imaging with color Doppler and spectral Doppler analysis, enabling simultaneous assessment of tissue morphology and vascular hemodynamics. While B-scan provides structural information on lesion size, shape, and internal reflectivity, the addition of Doppler techniques allows evaluation of blood flow within and around intraocular and orbital lesions. Color Doppler imaging visualizes the presence and spatial distribution of vascular flow, whereas spectral Doppler enables quantitative analysis of flow characteristics, including peak systolic velocity, end-diastolic velocity, and derived indices such as the resistive index. These parameters may provide indirect insight into vascular resistance and perfusion patterns, which can be relevant in differentiating pathological processes [20].

In the context of intraocular tumors, triplex ultrasonography may reveal intrinsic vascularity, often characterized by detectable internal blood flow and altered hemodynamic profiles. In contrast, inflammatory lesions may demonstrate more variable or diffuse vascular patterns, sometimes associated with surrounding hyperemia rather than organized internal circulation. However, significant overlap exists, and Doppler findings alone are rarely sufficient for definitive differentiation. Moreover, the small size of intraocular vessels, low flow velocities, and technical limitations related to probe positioning and angle dependency may reduce sensitivity, particularly in posterior segment lesions. Despite these limitations, triplex ultrasonography provides a valuable intermediate layer between structural imaging and biomechanical assessment. By adding a functional vascular dimension to conventional ultrasound, it complements both morphological evaluation and emerging techniques such as elastography. Within a multimodal imaging framework, Doppler-derived hemodynamic information can contribute to pattern recognition and improve diagnostic confidence when interpreted in conjunction with structural and biomechanical findings [17,20].

### 3.4. Ultrasound Elastography

Ultrasound elastography is based on the fundamental observation that pathological tissues differ from normal tissues not only in morphology but also in their mechanical properties. What was historically assessed qualitatively through palpation can now be evaluated quantitatively by measuring tissue deformation or wave propagation in response to applied force [21,22]. In this way, elastography introduces a biomechanical dimension to imaging that complements conventional grayscale and Doppler ultrasound. Two principal approaches are currently used: strain elastography (SE) and shear wave elastography (SWE). Although often grouped together, they rely on distinct physical principles and provide different types of information. SE measures tissue deformation in response to external or endogenous stress, expressing stiffness as a relative parameter within the field of view [23]. Because it depends on applied compression, SE is inherently operator-dependent and subject to variability, particularly in deformable structures such as the eye, where even minimal probe pressure can alter local stress distribution. As a result, SE is best regarded as a qualitative or comparative technique rather than a fully quantitative method [24]. In contrast, SWE generates shear waves within the tissue using acoustic radiation force and tracks their propagation in real time. Since shear wave velocity correlates with tissue stiffness, SWE provides quantitative measurements, typically expressed in meters per second or converted to Young’s modulus [25]. This wave-based approach enables more reproducible comparisons across tissues, time points, and patients, provided that acquisition conditions are standardized [21,24,26]. Despite its quantitative nature, the interpretation of SWE in ocular applications requires caution. The conversion of shear wave velocity into elasticity assumes that tissues behave as linear, isotropic, and homogeneous materials—conditions that are not met in the eye [27]. Ocular tissues are layered, anisotropic, and viscoelastic, with complex interfaces between structures such as the sclera, choroid, retina, and tumor tissue. In addition, the small size of intraocular lesions relative to the ultrasound beam introduces partial volume effects and boundary artifacts. Consequently, measured values should be interpreted as effective or relative indicators of stiffness rather than absolute material constants [24].

A further distinction lies between static and dynamic mechanical responses. SE reflects tissue deformation under quasi-static loading, whereas SWE captures the propagation of transient shear waves, which are influenced not only by elasticity but also by viscosity and structural heterogeneity [28]. In biological tissues, particularly tumors, stiffness is often spatially heterogeneous and influenced by factors such as cellular density, extracellular matrix composition, vascular architecture, and interstitial pressure [29]. These considerations are particularly relevant in intraocular imaging, where anatomical and technical constraints further complicate measurements. The globe is a closed, fluid-filled structure in which small changes in globe position or patient motion may affect results. Moreover, steep stiffness gradients between adjacent tissues (e.g., vitreous, retina, sclera) can distort shear wave propagation [30]. Careful attention to acquisition technique, region-of-interest placement, and reproducibility is therefore essential. Overall, SWE represents a significant advancement by enabling quantitative assessment of tissue biomechanics. However, in ophthalmic applications, its greatest value lies in identifying relative differences and biomechanical patterns rather than relying on absolute numerical thresholds, particularly in complex scenarios such as differentiating tumors from inflammatory lesions. Nevertheless, it is important to note that although SWE does not depend on operator-induced tissue compression, unlike strain elastography, excessive probe pressure during ocular examination may indirectly influence measurements by transiently altering globe geometry, increasing intraocular pressure, or modifying region-of-interest positioning. Therefore, careful probe handling remains essential for reproducible acquisitions.

#### 3.4.1. Rationale for Elastography in Tumor–Inflammation Differentiation

The potential role of elastography in distinguishing intraocular tumors from inflammatory lesions is focused in fundamental differences in tissue biomechanics. Malignant tumors typically demonstrate increased stiffness due to high cellular density, extracellular matrix remodeling, fibrosis, and altered vascular architecture [31]. In contrast, inflammatory processes may exhibit variable stiffness depending on disease stage, ranging from soft, edematous tissue in acute phases to increased rigidity in chronic fibrotic states [24,32].

In ophthalmology, differentiating intraocular tumors, such as uveal melanoma, metastasis, or lymphoma, from inflammatory conditions like posterior scleritis or granulomatous disease remains challenging, particularly in atypical presentations. Conventional imaging modalities (B-scan ultrasonography, OCT, angiography) provide essential morphological and vascular information but do not directly assess tissue biomechanics [17]. Elastography introduces a complementary parameter by enabling non-invasive “digital palpation” of intraocular lesions. As highlighted in recent clinical work, SWE allows quantitative assessment of tissue stiffness within both tumoral and peritumoral regions, offering insights into lesion composition and structural organization [33]. Importantly, rigidity alone is unlikely to serve as a sole discriminator between tumor and inflammation. Instead, its diagnostic value lies in identifying biomechanical patterns such as focal stiffness, internal heterogeneity, and perilesional gradients which, when combined with structural and vascular imaging, may improve diagnostic confidence. This enhances the concept that elastography should be incorporated within a multi-modality fusion framework, rather than used in isolation [24].

#### 3.4.2. Current Evidence and Translational Experience for Ocular Ultrasound Elastography

The application of SWE in intraocular imaging remains in its early stages, with most available data derived from feasibility studies and investigations of ocular and periocular structures. Early work demonstrated that ultrasound elastography can detect biomechanical differences across ocular tissues, including the retina–choroid–sclera complex, vitreous body, extraocular muscles, and optic nerve, suggesting a potential role in characterizing disease-related structural changes [24].

The most compelling evidence to date in ocular oncology comes from a recent prospective clinical study evaluating SWE in choroidal melanoma. In this study, tumor stiffness values were significantly higher than those of adjacent choroid and orbital fat (mean tumor stiffness approximately 34.6 kPa versus 9.4 kPa and 7.0 kPa, respectively), indicating that melanomas exhibit a distinct biomechanical profile compared to surrounding tissues [33]. Furthermore, tumor stiffness demonstrated significant correlations with both tumor height and intratumoral resistive index, suggesting an association between biomechanical properties, tumor morphology, and vascular resistance. These findings support the hypothesis that SWE-derived parameters may reflect not only structural characteristics but also aspects of tumor biology and aggressiveness [33,34]. Evidence from other ocular conditions further supports the feasibility of elastography in detecting disease-related biomechanical alterations. Increased stiffness has been reported in glaucomatous optic nerves and fibrotic extraocular muscles, indicating that both neoplastic and non-neoplastic processes can be characterized by altered tissue elasticity [24].

However, several limitations must be acknowledged. Current studies are limited by small sample sizes, lack of standardized acquisition protocols, and absence of histopathological correlation. Additionally, overlap in stiffness values between different pathological entities may reduce specificity [24,35,36,37]. Consequently, SWE should currently be regarded as a promising adjunctive modality rather than a definitive diagnostic tool.

#### 3.4.3. Technical Considerations and Safety in Ocular Ultrasound Elastography

The application of SWE in intraocular imaging presents unique technical and safety challenges. The eye is particularly susceptible to ultrasound-induced bioeffects due to its delicate structures and limited capacity for heat dissipation. As a result, strict adherence to safety guidelines is essential [38,39]. For ocular ultrasound safety, it needs to be considered that advanced ultrasound modalities, including Doppler and SWE, may generate acoustic outputs exceeding recommended limits for ophthalmic applications and should therefore be used with caution, or considered experimental in certain contexts [38]. Key safety parameters include the thermal index (TI) and mechanical index (MI), which should be maintained within recommended thresholds (typically TI ≤ 1.0 and MI ≤ 0.23) in accordance with the ALARA (As Low As Reasonably Achievable) principle. In the choroidal melanoma study, these limits are to be respected, and examination time should be minimized to reduce potential risk [33]. Given the particular susceptibility of ocular tissues to ultrasound-induced bioeffects, strict adherence to established ophthalmic ultrasound safety recommendations is mandatory during SWE examinations. These considerations are especially relevant when SWE is performed on radiology-grade ultrasound systems, where acoustic output settings may differ from those of dedicated ophthalmic ultrasound equipment.

From a technical perspective, several factors may influence SWE measurements, including probe pressure and positioning (which may alter intraocular pressure and tissue deformation), eye motion and pulsatility (introducing variability in measurements), small lesion size and anatomical complexity (complicating region-of-interest placement) and tissue anisotropy and heterogeneity (limiting the validity of simplified elasticity models). Moreover, elastography measurements are not yet standardized across different ultrasound platforms, limiting inter-study comparability [40]. Future developments should focus on reducing acoustic output, improving spatial resolution, and integrating elastography with other imaging modalities such as OCT and Doppler ultrasound [41]. Such advancements are essential for the safe and reliable incorporation of SWE into routine ophthalmic practice. Moreover, although shear wave elastography is a well-established quantitative ultrasonographic technique with broad clinical applications in multiple organ systems, its application to intraocular imaging remains at an early stage, and the available ophthalmic evidence is currently limited to relatively small feasibility and pilot studies.

## 4. Multi-Modality Imaging Fusion

### 4.1. Concept of Imaging Fusion in Ophthalmology

Multi-modality imaging fusion refers to the integration of data acquired from different imaging techniques into a unified interpretative framework, allowing simultaneous assessment of complementary tissue properties. In ophthalmology, this approach addresses a fundamental limitation of individual imaging modalities: each technique provides information from a specific perspective—structural, vascular, or functional—yet no single modality is sufficient to fully characterize complex intraocular pathology [42]. Traditionally, clinicians mentally integrate findings from various imaging studies, such as OCT, angiography, and ultrasonography. However, this subjective synthesis is inherently limited by cognitive bias and variability in interpretation. Imaging fusion aims to overcome these limitations by either spatially co-registering images from different modalities or by conceptually integrating their outputs into a structured diagnostic model. Apart from dedicated ophthalmic imaging modalities, image fusion has been previously applied in ophthalmic oncology for transverse radiological imaging, such as fusion between MRI and CT scans to enhance the accuracy of Ru-106 radioactive plaque placement in ophthalmic brachytherapy procedures [43].

It is important to distinguish between two complementary concepts of multimodal imaging. The first is *technical image fusion*, in which images acquired by different modalities are spatially co-registered or digitally overlaid to produce a single composite image. This approach preserves anatomical correspondence between datasets and is increasingly used in image-guided interventions and treatment planning. The second is *conceptual multimodal integration*, whereby information obtained from different imaging modalities is interpreted collectively to generate a more comprehensive assessment of tissue characteristics, even in the absence of physical image registration. The diagnostic framework proposed in this review primarily reflects this latter concept, while recognizing that future advances in image co-registration technologies may enable more sophisticated fusion strategies in ophthalmology.

In the context of intraocular imaging, the need for such integration is particularly evident in cases where inflammatory and neoplastic processes overlap clinically and radiologically. Both entities may share features such as mass effect, subretinal fluid, or vascular leakage, making differentiation challenging when relying on a single imaging modality. Fusion imaging enables a more comprehensive characterization by combining microstructural detail (OCT), vascular dynamics (FFA, ICGA, OCT-A), and, more recently, biomechanical properties (elastography). Thus, imaging fusion should not be viewed merely as a technical process of image overlay, but rather as a paradigm shift toward multidimensional tissue characterization [42].

**Figure 1:**
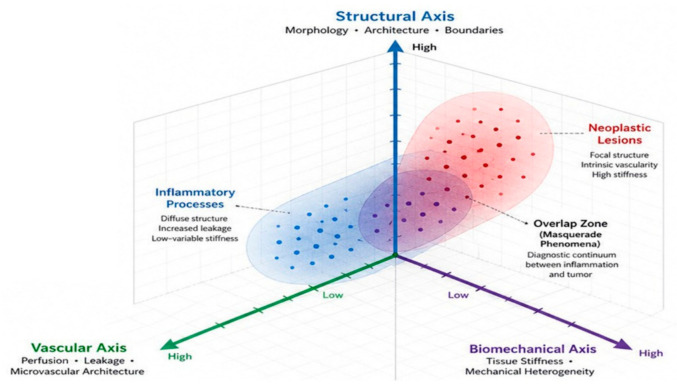
Conceptual (non-validated) tri-axis framework illustrating the integration of structural, vascular, and biomechanical imaging information during multimodal assessment of intraocular disease. The model is intended to facilitate clinical interpretation rather than represent a validated diagnostic algorithm. The model integrates structural, vascular, and biomechanical imaging domains to characterize intraocular lesions. Inflammatory processes are associated with diffuse structural changes, increased leakage, and relatively low or variable stiffness, whereas neoplastic lesions tend to demonstrate focal organization, intrinsic vascularity, and increased stiffness. The overlap zone illustrates the diagnostic continuum of masquerade phenomena, where inflammatory and neoplastic entities may share overlapping imaging characteristics.

To conceptually illustrate the integration of multimodal imaging, we propose a tri-axis framework (Figure 1). This framework is intended as a heuristic model for integrating structural, vascular, and biomechanical information rather than as a clinically validated diagnostic algorithm. The potential value of this approach lies in facilitating recognition of patterns of concordance across all three axes rather than relying on isolated imaging findings. Future clinical studies are required to validate its diagnostic performance and reproducibility. In this framework, intraocular lesion characterization is represented within a three-dimensional aspace defined by structural, vascular, and biomechanical domains. Each axis corresponds to a distinct but complementary dimension of tissue assessment. The structural axis incorporates modalities such as optical coherence tomography (OCT), B-scan ultrasonography, and ultra-widefield imaging, which together define lesion morphology, boundaries, and spatial organization. The vascular axis includes fluorescein angiography (FFA), indocyanine green angiography (ICGA), and OCT angiography (OCT-A), providing information on perfusion, leakage, and microvascular architecture. The biomechanical axis is defined by elastography techniques, including shear wave elastography (SWE), strain elastography (SE), and emerging OCT-based elastography, reflecting tissue stiffness and mechanical heterogeneity.

Within this multidimensional space, intraocular lesions can be positioned according to their composite imaging characteristics. Inflammatory processes tend to cluster toward regions characterized by diffuse structural changes, increased vascular leakage, and relatively low or variable stiffness [44]. In contrast, neoplastic lesions are more frequently associated with focal structural organization, intrinsic vascular networks, and increased tissue stiffness. Importantly, these regions are not mutually exclusive and may partially overlap, reflecting the well-recognized diagnostic continuum between inflammation and tumor (“masquerade” phenomena) [45]. The diagnostic value of the model lies in identifying patterns of concordance across all three axes rather than relying on isolated imaging findings.

### 4.2. Fusion of Structural, Vascular, and Biomechanical Data

A practical framework for multi-modality imaging fusion can be conceptualized along three principal axes: structure, vascularity, and biomechanics. Structural imaging modalities, primarily OCT and ultrasonography, provide high-resolution information on lesion morphology, anatomical location, and internal architecture. OCT excels in resolving retinal microstructure and identifying subtle layer-specific abnormalities, whereas B-scan ultrasonography allows visualization of deeper or optically inaccessible lesions, offering a broader anatomical context. Vascular imaging modalities, including fluorescein angiography, indocyanine green angiography, and OCT angiography, contribute dynamic and layer-specific information on blood flow, vascular permeability, and microvascular architecture. These techniques are particularly useful in assessing disease activity, identifying ischemia, and characterizing tumor-associated vasculature. However, vascular patterns alone are often insufficient for definitive differentiation, as both tumors and inflammatory lesions may demonstrate leakage, staining, or abnormal vascular networks. Biomechanical assessment, introduced through ultrasound elastography and emerging OCT-based techniques, adds a fundamentally different dimension by evaluating tissue stiffness and mechanical behavior. This “digital palpation” provides indirect insight into tissue composition, cellular density, and extracellular matrix organization. Malignant tumors tend to exhibit increased stiffness and internal heterogeneity, whereas inflammatory lesions may demonstrate variable or more diffuse mechanical profiles depending on disease stage.

The integration of these three domains enables recognition of multimodal patterns, rather than reliance on isolated findings. For example, a lesion demonstrating well-defined structural boundaries on OCT, intrinsic vascular networks with leakage on angiography and increased focal stiffness on elastography is more likely to represent a neoplastic process than an inflammatory one. Conversely, diffuse structural thickening, widespread vascular leakage, and relatively homogeneous or lower stiffness may favor an inflammatory etiology. Importantly, the value of fusion lies not in any single parameter but in the concordance or discordance between modalities, which may reveal diagnostic clues not apparent in isolation.

### 4.3. Clinical Scenarios Benefiting from Fusion Imaging

**Figure 2:**
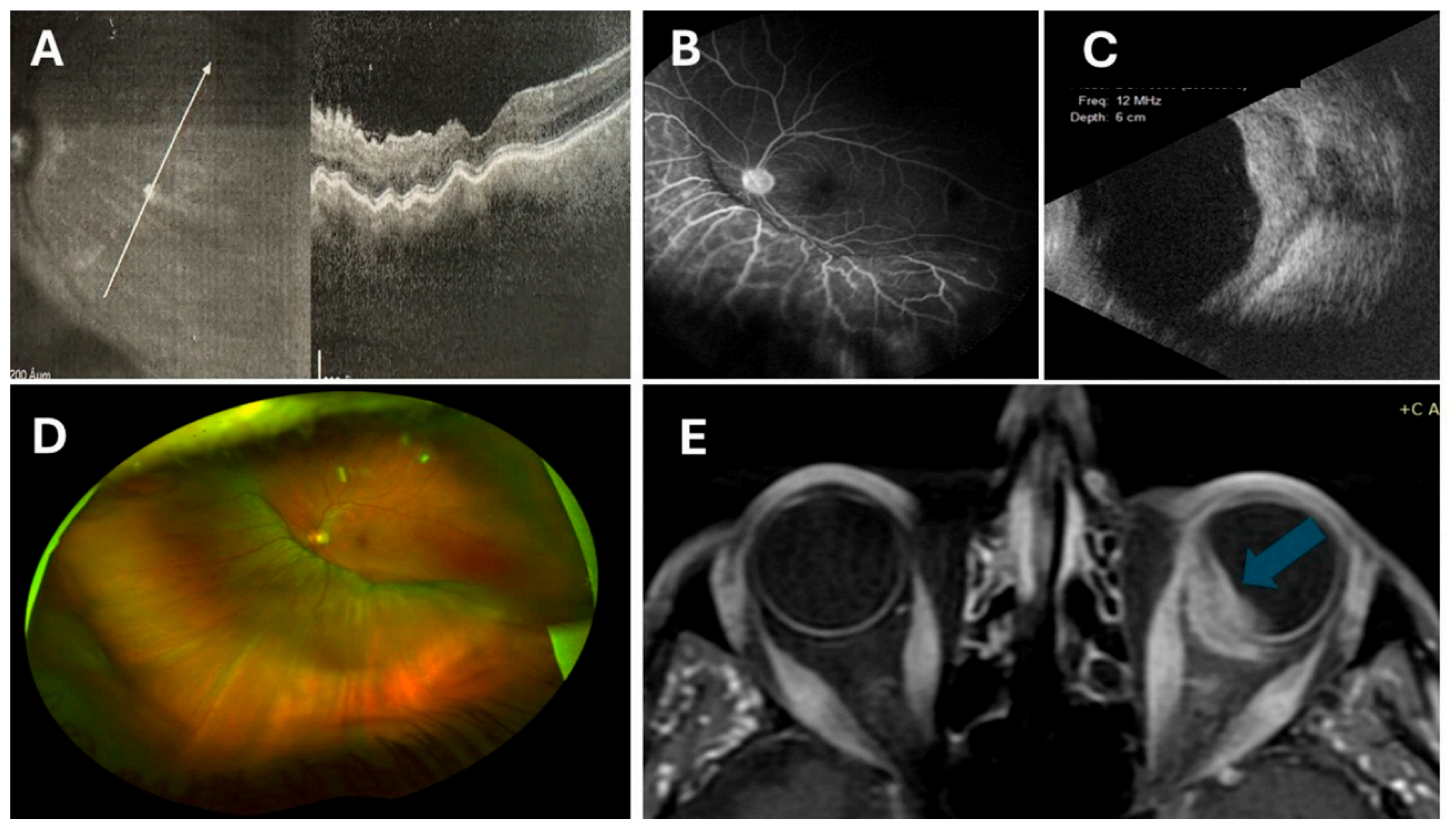
Composite imaging demonstrating structural, vascular, and radiological features of a bilateral intraocular lymphoproliferative lesion. (**A**) Optical coherence tomography (OCT) showing undulating choroidal thickening with overlying retinal changes (arrow highilights the level of OCT section). (**B**) Fluorescein angiography (FFA) revealing diffuse vascular leakage and optic disc hyperfluorescence. (**C**) B-scan ultrasonography demonstrating a hyperechoic choroidal mass with associated thickening. (**D**) Ultra-widefield fundus imaging illustrating diffuse, plaque-like choroidal infiltration with subtle color changes consistent with a “pseudotumoral” pattern. (**E**) Orbital magnetic resonance imaging (MRI) showing bilateral intraocular masses with evidence of extraocular extension, more prominent in the left eye (blue arrow). The multimodal findings highlight the complementary roles of structural (OCT, ultrasound), vascular (FFA), and cross-sectional radiological imaging (MRI) in characterizing intraocular lymphoma, supporting a fusion-based diagnostic approach for distinguishing infiltrative neoplastic processes from inflammatory mimickers.

Multi-modality imaging fusion is particularly valuable in clinical scenarios characterized by diagnostic ambiguity, where conventional approaches fail to provide definitive differentiation between tumor and inflammation. One such scenario involves posterior uveitis with mass-like lesions, where inflammatory granulomas, infectious lesions, or lymphoma may mimic choroidal tumors (Figure 2). In these cases, OCT may reveal subretinal fluid or retinal disruption, while angiography demonstrates leakage; however, these findings are often non-specific. The addition of elastography may provide critical information, as focal areas of increased stiffness or internal heterogeneity may raise suspicion for neoplastic infiltration.

Another important application is in the evaluation of amelanotic or atypical choroidal lesions, where classic clinical features of melanoma may be absent. Structural imaging may be inconclusive, and angiographic patterns may overlap with inflammatory or vascular lesions [46]. Fusion imaging allows correlation of subtle structural abnormalities with vascular behavior and biomechanical properties, potentially improving diagnostic confidence and reducing the need for invasive procedures. Fusion approaches are also beneficial in monitoring treatment response, particularly in distinguishing between active disease and residual structural changes. For example, reduction in vascular leakage on angiography combined with normalization of tissue stiffness may indicate therapeutic response, even when structural abnormalities persist on OCT. Conversely, persistent focal stiffness despite apparent clinical improvement may suggest underlying residual tumor or fibrosis. Finally, imaging fusion may play a role in guiding clinical decision-making, including the need for biopsy, initiation of immunosuppressive therapy, or oncologic intervention. By integrating multiple dimensions of tissue characterization, clinicians can achieve a more nuanced understanding of disease behavior, potentially improving both diagnostic confidence and patient outcomes [47].

## 5. Integrating Ophthalmic Ultrasound Elastography into a Multimodal Diagnostic Framework

**Figure 3:**
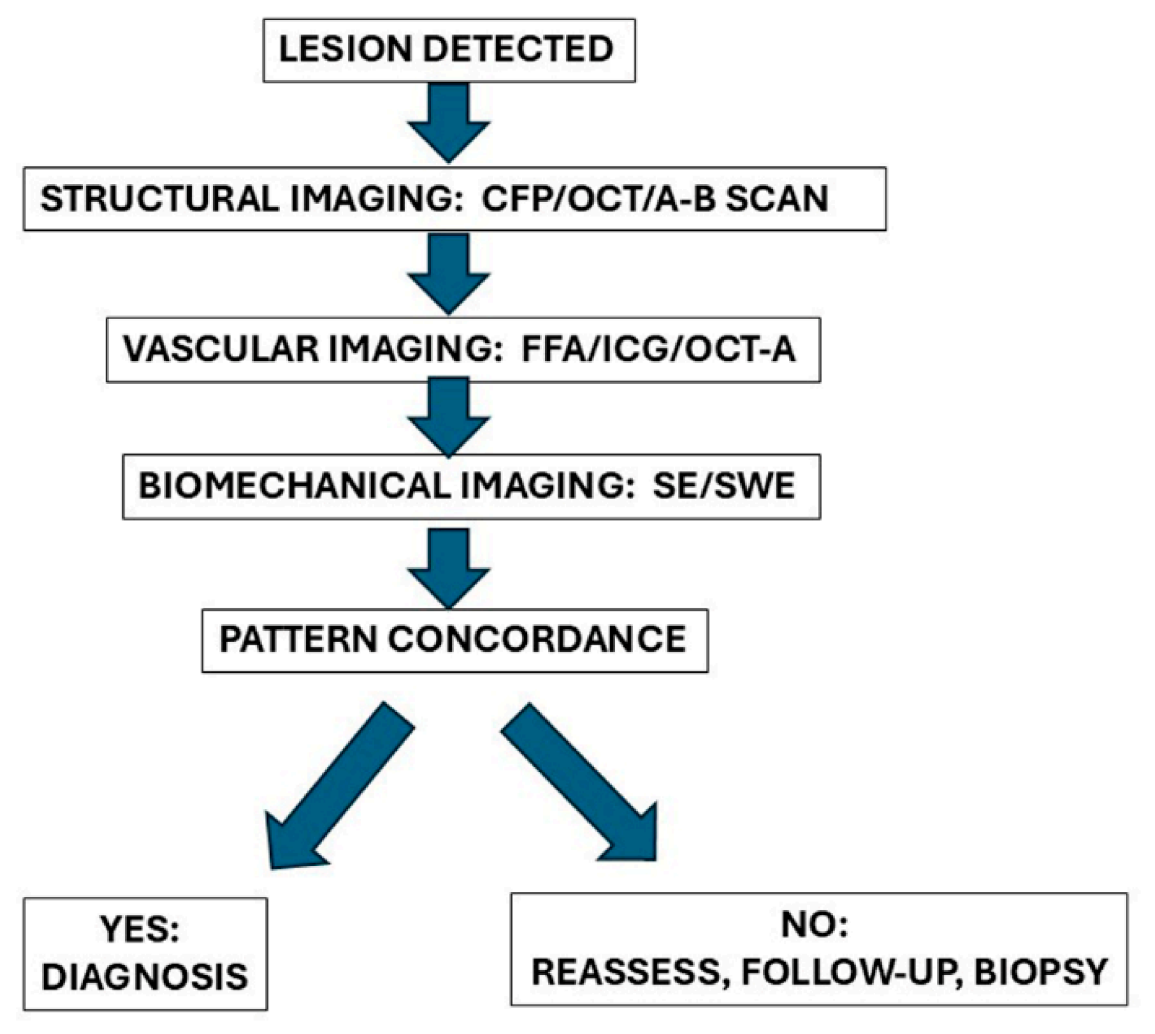
Conceptual workflow illustrating multimodal integration in the evaluation of suspicious intraocular lesions. Structural, vascular, and biomechanical imaging findings are interpreted collectively to assess the concordance of multimodal information. Concordant findings may increase diagnostic confidence and support a working diagnosis, whereas discordant or inconclusive findings should prompt reassessment, additional imaging, follow-up, or tissue biopsy when clinically indicated. This workflow is intended as a conceptual clinical framework to facilitate multimodal interpretation and should not be regarded as a validated diagnostic algorithm.

The integration of multi-modality imaging can be translated into a practical diagnostic workflow aimed at improving differentiation between intraocular tumors and inflammatory conditions (Figure 3).

### 5.1. Step 1: Structural Assessment

Initial evaluation is based on structural imaging, primarily OCT and B-scan ultrasonography. Key features include lesion localization, definition of borders, internal reflectivity, and presence of associated findings such as subretinal fluid or retinal disruption. Well-circumscribed, elevated lesions favor a neoplastic process, whereas diffuse thickening or poorly defined boundaries may suggest inflammation (Figure 3).

### 5.2. Step 2: Vascular Characterization

Vascular imaging with fluorescein angiography, indocyanine green angiography, or OCT angiography is used to assess perfusion, leakage, and vascular architecture. Tumors often demonstrate intrinsic vascular networks and patterned leakage, whereas inflammatory lesions are more likely to show diffuse leakage, vasculitis, or capillary non-perfusion.

### 5.3. Step 3: Biomechanical Evaluation

Elastography is then incorporated to assess tissue stiffness. Increased focal stiffness and internal heterogeneity support a neoplastic etiology, while lower or more homogeneous stiffness patterns are more consistent with inflammatory processes, although overlap exists.

### 5.4. Step 4: Multimodal Integration (Fusion Step)

Findings from all three domains are integrated to identify concordant patterns. Diagnostic confidence increases when structural, vascular, and biomechanical features align toward a single etiology. Discordant findings should prompt reconsideration, closer follow-up, or additional testing.

### 5.5. Step 5: Clinical Decision-Making

Based on the integrated assessment for concordant neoplastic features oncologic referral or biopsy should be considered. For concordant inflammatory features laboratory diagnostic work-up should be planned. And for indeterminate or discordant findings close monitoring or repeat imaging should be followed.

## 6. Future Trends and Perspectives

The continued evolution of multi-modality imaging in ophthalmology is expected to shift the field toward increasingly quantitative, integrative, and data-driven diagnostic approaches. Several emerging directions may further enhance the clinical utility of imaging fusion in differentiating intraocular tumors from inflammatory conditions.

AI-assisted fusion represents one of the most promising developments. Machine learning and deep learning algorithms have the potential to integrate heterogeneous imaging datasets—structural, vascular, and biomechanical—into unified predictive models. By identifying complex, non-linear relationships between imaging features, artificial intelligence (AI) systems may potentially assist human interpretation in the diagnostic process. In particular, AI-driven pattern recognition could facilitate automated classification of lesions within the proposed tri-axis framework, reducing interobserver variability and enabling real-time clinical decision support [47].

Quantitative stiffness thresholds derived from elastography may further refine diagnostic specificity. While current applications of shear wave elastography (SWE) rely primarily on relative comparisons and pattern recognition, future studies may establish reproducible stiffness ranges associated with specific pathological entities. The development of standardized acquisition protocols and calibration across imaging platforms will be essential to enable meaningful comparisons and define clinically actionable thresholds. However, given the complex and heterogeneous nature of ocular tissues, such thresholds are likely to be probabilistic rather than absolute [40,48].

Longitudinal monitoring using multimodal imaging fusion offers another important avenue for clinical application. Rather than relying on single time-point assessments, repeated measurements across structural, vascular, and biomechanical domains may provide insight into disease progression, treatment response, and early relapse. Changes in tissue stiffness, vascular activity, or structural organization over time may serve as dynamic biomarkers, allowing earlier detection of therapeutic success or failure compared to conventional imaging alone. Finally, multicenter validation will be critical for translating these approaches into routine clinical practice. Current evidence for ocular elastography and imaging fusion remains limited to small-scale or single-center studies. Larger, prospective, multicenter investigations are needed to validate diagnostic performance, establish standardized protocols, and assess reproducibility across different populations and imaging systems. Such collaborative efforts will also be essential for generating robust datasets required for AI training and validation.

Taken together, these developments suggest a transition from qualitative, modality-specific interpretation toward a quantitative, integrated, and longitudinal imaging paradigm. Within this evolving framework, multi-modality imaging fusion—augmented by artificial intelligence and standardized quantitative metrics—has the potential to assist in the clinical management of complex intraocular diseases.

## 7. Conclusions

Intraocular tumors and inflammatory conditions continue to represent a significant diagnostic challenge in ophthalmology, particularly in cases where clinical and imaging findings overlap within the spectrum of so-called masquerade syndromes. While advances in imaging have substantially improved the non-invasive evaluation of ocular pathology, no single modality is sufficient to reliably distinguish between these entities in all cases. This review highlights the complementary roles of structural, vascular, and biomechanical imaging in addressing this diagnostic dilemma. Conventional modalities such as OCT, angiography, and ultrasonography provide essential information on morphology and vascular behavior, yet they do not directly capture tissue mechanical properties. Ultrasound elastography, particularly SWE, introduces a novel biomechanical dimension, enabling quantitative assessment of tissue stiffness and offering additional insight into lesion composition and organization. The integration of these modalities within a multi-dimensional framework represents a conceptual shift from modality-based interpretation toward pattern-based diagnosis. The proposed tri-axis model of imaging fusion provides a structured approach to combining structural, vascular, and biomechanical data, facilitating recognition of multimodal signatures associated with inflammatory and neoplastic processes. Importantly, the diagnostic value lies not in any single parameter but in the concordance of findings across domains. From a clinical perspective, the incorporation of elastography into a multimodal workflow has the potential to improve diagnostic confidence, guide management decisions, and reduce reliance on invasive procedures such as biopsy. However, although SWE represents a well-established quantitative ultrasonographic technique, its application to intraocular disease remains an emerging field of investigation. At present, SWE should be regarded as a complementary imaging modality that provides biomechanical information not available from conventional structural or vascular imaging techniques. Its greatest potential lies in its integration within a multimodal diagnostic framework, where biomechanical, structural, and vascular findings can be interpreted collectively to improve diagnostic confidence. However, prospective multicenter studies, standardized acquisition protocols, and validation against established clinical and histopathological reference standards are still required before routine clinical implementation can be recommended.

## Data Availability

No new data were created or analyzed in this study. Data sharing is not applicable to this article.

## References

[1] Tsai T., O’Brien J.M. (2002). Masquerade syndromes: Malignancies mimicking inflammation in the eye. Int. Ophthalmol. Clin..

[2] Rothova A., Ooijman F., Kerkhoff F., Van der Lelij A., Lokhorst H.M. (2001). Uveitis masquerade syndromes. Ophthalmology.

[3] Dimaras H., Corson T.W., Cobrinik D., White A., Zhao J., Munier F.L., Abramson D.H., Shields C.L., Chantada G.L., Njuguna F. (2015). Retinoblastoma. Nat. Rev. Dis. Prim..

[4] Martel A., Baillif S., Nahon-Esteve S., Gastaud L., Bertolotto C., Roméo B., Mograbi B., Lassalle S., Hofman P. (2020). Liquid biopsy for solid ophthalmic malignancies: An updated review and perspectives. Cancers.

[5] Fogel-Levin M., Sadda S.R., Rosenfeld P.J., Waheed N., Querques G., Freund B.K., Sarraf D. (2022). Advanced retinal imaging and applications for clinical practice: A consensus review. Surv. Ophthalmol..

[6] Yannuzzi L.A., Ober M.D., Slakter J.S., Spaide R.F., Fisher Y.L., Flower R.W., Rosen R. (2004). Ophthalmic fundus imaging: Today and beyond. Am. J. Ophthalmol..

[7] Samuelsson D., Sznage M., Engelsberg K., Wittström E. (2016). Clinical, optical coherence tomography, and fundus autofluorescence findings in patients with intraocular tumors. Clin. Ophthalmol..

[8] Mueller A.J., Freeman W.R., Folberg R., Bartsch D.U., Scheider A., Schaller U., Kampik A. (1999). Evaluation of microvascularization pattern visibility in human choroidal melanomas: Comparison of confocal fluorescein with indocyanine green angiography. Graefes Arch. Clin. Exp. Ophthalmol..

[9] Lee J., Sagong M. (2016). Ultra-widefield retina imaging: Principles of technology and clinical applications. J. Retin..

[10] Shoughy S.S., Arevalo J.F., Kozak I. (2015). Update on wide-and ultra-widefield retinal imaging. Indian J. Ophthalmol..

[11] Say E.A.T., Shah S.U., Ferenczy S., Shields C.L. (2011). Optical coherence tomography of retinal and choroidal tumors. J. Ophthalmol..

[12] Chen C.-L., Wang R.K. (2017). Optical coherence tomography based angiography. Biomed. Opt. Express.

[13] Greig E.C., Duker J.S., Waheed N.K. (2020). A practical guide to optical coherence tomography angiography interpretation. Int. J. Retin. Vitr..

[14] Zheng S., Bai Y., Xu Z., Liu P., Ni G. (2021). Optical coherence tomography for three-dimensional imaging in the biomedical field: A review. Front. Phys..

[15] Javed A., Khanna A., Palmer E., Wilde C., Zaman A., Orr G., Kumudhan D., Lakshmanan A., Panos G.D. (2023). Optical coherence tomography angiography: A review of the current literature. J. Int. Med. Res..

[16] Kirby M.A., Pelivanov I., Song S., Ambrozinski Ł., Yoon S.J., Gao L., Li D., Shen T.T., Wang R.K., O’Donnell M. (2017). Optical coherence elastography in ophthalmology. J. Biomed. Opt..

[17] Kadakia A., Zhang J., Yao X., Zhou Q., Heiferman M.J. (2023). Ultrasound in ocular oncology: Technical advances, clinical applications, and limitations. Exp. Biol. Med..

[18] Silverman R.H. (2009). High-resolution ultrasound imaging of the eye–a review. Clin. Exp. Ophthalmol..

[19] Nanji A.A., Mercado C., Galor A., Dubovy S., Karp C.L. (2017). Updates in ocular surface tumor diagnostics. Int. Ophthalmol. Clin..

[20] Modrzejewska M. (2019). Guidelines for ultrasound examination in ophthalmology. Part III: Color Doppler ultrasonography. J. Ultrason..

[21] Garra B.S. (2015). Elastography: History, principles, and technique comparison. Abdom. Imaging.

[22] Walker H.K. (1990). The origins of the history and physical examination. Clinical Methods: The History, Physical, and Laboratory Examinations.

[23] Cui X.W., Li K.N., Yi A.J., Wang B., Wei Q., Wu G.G., Dietrich C.F. (2022). Ultrasound elastography. Endosc. Ultrasound.

[24] Bontzos G., Douglas V.P., Douglas K.A.A., Kapsala Z., Drakonaki E.E., Detorakis E.T. (2021). Ultrasound elastography in ocular and periocular tissues: A review. Curr. Med. Imaging Rev..

[25] Kim H., Kim H., Han B.K., Choi J.S., Ko E.S., Ko E.Y. (2022). Accuracy and reproducibility of shear wave elastography according to the size and elasticity of lesions: A phantom study. Medicine.

[26] Woo J.H., Ko E.Y., Han B.K. (2021). Comparison of 2 shear wave elastography systems in reproducibility and accuracy using an elasticity phantom. Medicine.

[27] Osika M., Kijanka P. (2024). Ultrasound shear wave propagation modeling in general tissue–like viscoelastic materials. Ultrasound Med. Biol..

[28] Rudenko O.V., Sarvazyan A.P. (2014). Wave anisotropy of shear viscosity and elasticity. Acoust. Phys..

[29] Civale J., Parasaram V., Bamber J.C., Harris E.J. (2022). High frequency ultrasound vibrational shear wave elastography for preclinical research. Phys. Med. Biol..

[30] Dubinsky T.J., Shah H.U., Erpelding T.N., Sannananja B., Sonneborn R., Zhang M. (2019). Propagation imaging in the demonstration of common shear wave artifacts. J. Ultrasound Med..

[31] Paolillo M., Schinelli S. (2019). Extracellular matrix alterations in metastatic processes. Int. J. Mol. Sci..

[32] Dietrich C.F., Jenssen C., Arcidiacono P.G., Cui X.W., Giovannini M., Hocke M., Iglesias-Garcia J., Saftoiu A., Sun S., Chiorean L. (2015). Endoscopic ultrasound: Elastographic lymph node evaluation. Endosc. Ultrasound.

[33] Douglas K.A.A., Drakonaki E.E., Douglas V.P., Detorakis E.T. (2024). Shear-wave elastographic imaging in choroidal melanomas: Clinical and hemodynamic correlations. Jpn. J. Ophthalmol..

[34] Wildeboer R.R., Mannaerts C.K., van Sloun R.J., Budäus L., Tilki D., Wijkstra H., Salomon G., Mischi M. (2020). Automated multiparametric localization of prostate cancer based on B-mode, shear-wave elastography, and contrast-enhanced ultrasound radiomics. Eur. Radiol..

[35] Bruce M., Kolokythas O., Ferraioli G., Filice C., O’Donnell M. (2017). Limitations and artifacts in shear-wave elastography of the liver. Biomed. Eng. Lett..

[36] Franchi-Abella S., Elie C., Correas J.M. (2013). Ultrasound elastography: Advantages, limitations and artefacts of the different techniques from a study on a phantom. Diagn. Interv. Imaging.

[37] Ooi C.-C., Malliaras P., Schneider M.E., Connell D.A. (2014). “Soft, hard, or just right?” Applications and limitations of axial-strain sonoelastography and shear-wave elastography in the assessment of tendon injuries. Skelet. Radiol..

[38] Abramowicz J.S., Adhikari S., Dickman E., Estroff J.A., Harris G.R., Nomura J., Silverman R.H., Taylor L.A., Barr R.G. (2022). Ocular ultrasound: Review of bioeffects and safety, including fetal and point-of-care perspective. J. Ultrasound Med..

[39] Shankar H., Pagel P.S., Warner D.S. (2011). Potential adverse ultrasound-related biological effects: A critical review. Anesthesiology.

[40] Sigrist R.M.S., Liau J., El Kaffas A., Chammas M.C., Willmann J.K. (2017). Ultrasound elastography: Review of techniques and clinical applications. Theranostics.

[41] Kloth C., Kratzer W., Schmidberger J., Beer M., Clevert D.A., Graeter T. (2021). Ultrasound 2020–Diagnostics & Therapy: On the Way to Multimodal Ultrasound: Contrast-Enhanced Ultrasound (Ceus), Microvascular Doppler Techniques, Fusion Imaging, Sonoelastography, Interventional Sonography.

[42] El-Ateif S., Idri A. (2024). Multimodality fusion strategies in eye disease diagnosis. J. Imaging Inform. Med..

[43] Detorakis E.T., Perisinakis K., Drakonaki E., Liakopoulos D., Tzedakis A., Papadaki E., Tsilimbaris M.K. (2021). MRI and dual-energy CT fusion anatomic imaging in Ru-106 ophthalmic brachytherapy. Brachytherapy.

[44] Mahaling B., Low S.W., Beck M., Kumar D., Ahmed S., Connor T.B., Ahmad B., Chaurasia S.S. (2022). Damage-associated molecular patterns (DAMPs) in retinal disorders. Int. J. Mol. Sci..

[45] Mahajan A., Crum A., Johnson M.H., Materin M.A. (2011). Ocular Neoplastic Disease.

[46] Pichi F., Roberts P., Neri P. (2019). The broad spectrum of application of optical coherence tomography angiography to the anterior segment of the eye in inflammatory conditions: A review of the literature. J. Ophthalmic Inflamm. Infect..

[47] Swaminathan U., Daigavane S. (2024). Unveiling the potential: A comprehensive review of artificial intelligence applications in ophthalmology and future prospects. Cureus.

[48] Pelea M.-A., Șerban O., Bădărînză M., Fodor D. (2025). Advancements and challenges in Shear-Wave Elastography of tendons: A comprehensive review. Med. Ultrason..

